# CHAC2, downregulated in gastric and colorectal cancers, acted as a tumor suppressor inducing apoptosis and autophagy through unfolded protein response

**DOI:** 10.1038/cddis.2017.405

**Published:** 2017-08-24

**Authors:** Shuiping Liu, Weiqiang Fei, Qinglan Shi, Qiang Li, Yeye Kuang, Chan Wang, Chao He, Xiaotong Hu

**Affiliations:** 1 Biomedical Research Center and Key Laboratory of Biotherapy of Zhejiang Province, Sir Run Run Shaw Hospital, Zhejiang University, Hangzhou, China

## Abstract

Tumor suppressor genes play a key role in cancer pathogenesis. Through massive expression profiling we identified CHAC2 as a frequently downregulated gene in gastric and colorectal cancers. Immunohistochemistry and western blot revealed that CHAC2 was downregulated in most tumor tissues, and 3-year survival rate of patients with high CHAC2 expression was significantly higher than that of patients with low CHAC2 expression (*P*<0.001 and *P*=0.001, respectively). The data of univariate analysis and multivariate analysis suggested that CHAC2 could serve as an independent prognostic marker. Our results showed for the first time that CHAC2 was degraded by the ubiquitin-proteasome pathway and CHAC2 expression inhibited tumor cell growth, proliferation, migration *in vitro* and *in vivo*. Mechanistic study showed that CHAC2 induced mitochondrial apoptosis and autophagy through unfolded protein response. So in gastric and colorectal cancer CHAC2 acted as a tumor suppressor and might have therapeutic implication for patients.

Gastric and colorectal cancers remain highly prevalent tumors, causing high morbidity and mortality in the world. Its molecular pathogenesis is believed to be a multistep process involving a number of genetic and epigenetic factors including the inactivation of tumor suppressor genes (TSGs).^1, 2^ Exploring and characterization of more novel TSGs will greatly facilitate to develop novel effective individualized therapeutic strategies.

We searched for candidate TSGs in digestive tumors through massive expression profiling, and found that CHAC2 was a frequently downregulated gene in gastric and colorectal cancers. CHAC2 is a member of cation transport regulator-like protein (CHAC) family which contain the BtrG/*γ*-glutamyl cyclotransferase (*γ*-GCT) fold and belong to the family of *γ*-GCTs. CHAC family members are reported to function as *γ*-GCT acting specifically to degrade glutathione but no other *γ*-glutamyl peptides using *in vivo* studies and novel *in vitro* assays.^3, 4^ The CHAC family is conserved across all phyla and represents the first cytosolic pathway for glutathione degradation in mammalian cells. Until now, two CHAC members, CHAC1 and CHAC2, have been identified in human genome. As we know, few literatures about CHAC2 have been reported, while there are several reports about CHAC1. CHAC1 overexpression enhanced apoptosis with an increase in TUNEL staining, cleaved PARP, and AIF nuclear translocation.^3^ Later, Kumar *et al.*^4^ reported that overexpression of mouse wild-type CHAC1 but not its catalytically inactive mutant (E4Q) led to glutathione depletion and enhanced apoptosis which could be reversed by addition of glutathione in yeasts. Overexpression of human CHAC1 also led to a robust depletion of glutathione in HEK293 cells.^5^ Moreover, Goebel *et al.*^6^ reported that elevated mRNA expression of CHAC1 splicing variants is associated with poor outcome for breast and ovarian cancer patients.

CHAC family members function as *γ*-GCT acting specifically to degrade glutathione, followed by disturbing the oxidation reduction potential of cell.^7^ Some pathophysiological conditions including disturbing the oxidation reduction potential of cell, known as endoplasmic reticulum (ER) stress, disrupt ER function and cause the accumulation of unfolded and/or misfolded proteins in the ER.^8^ ER stress leads to the activation of an adaptive signaling program, known as the ER stress response or unfolded protein response (UPR), which is mediated by three ER resident stress sensors, including PERK, IRE1, and ATF6.^9^

In a preliminary study, we found that CHAC2, an important member of CHAC family, was downregulated and might be a candidate TSG in gastrointestinal cancer. The role of CHAC2 in cancer progression is poorly explored. So in this study, we investigated the regulation mechanism of CHAC2 expression and explored its functions and signaling pathway in the development of gastric and colorectal cancers.

## Results

### Expression of CHAC2 was downregulated in cancer cell lines

**Figure 1:**
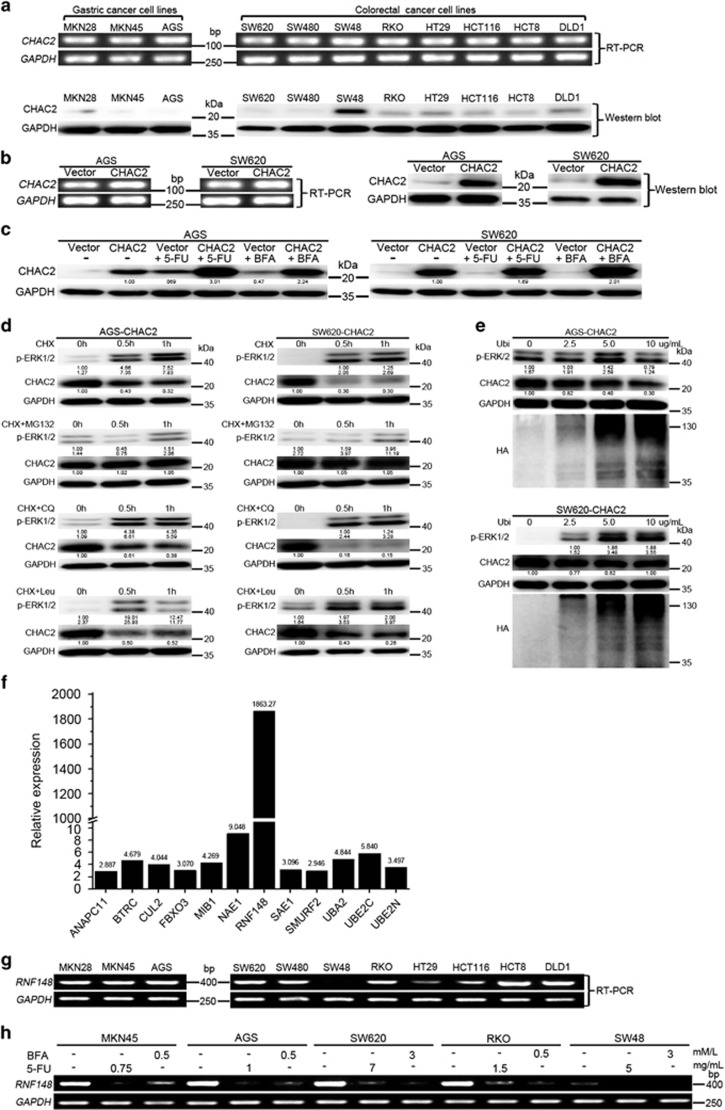
Analysis of CHAC2 expression and its degradation pathway in gastric and colorectal cancer cell lines. (**a**) The expression of CHAC2 in gastric and colorectal cancer cell lines was determined by RT-PCR and western bolt, using GAPDH as a control. (**b**) CHAC2 expression in stably transfected cells confirmed by RT-PCR and western blot. (**c**) Evaluation of CHAC2 expression after treatment of 5-FU or BFA. (**d**) Analysis of the potential degradation pathway of CHAC2 using different inhibitors. AGS-CHAC2 and SW620-CHAC2 cells were treated with 20 *μ*g/ml CHX with or without MG-132 (20 *μ*M) or Leu (100 *μ*M) or CQ (50 mM) in a time course. Cell lysates were subjected to western blot with primary antibodies including CHAC2, p-ERK and GAPDH. (**e**) The effect of ubiquitin on CHAC2 expression. AGS-CHAC2 and SW620-CHAC2 cells were incubated with indicated rhHA-ubiquitin for 12 h, and cell lysates were subjected to western blot with primary antibodies including CHAC2, HA tag andGAPDH. (**f**) 12 maximal upregulated genes checked by the Human Ubiquitination Pathway RT^2^ Profiler PCR Array were shown in the bar graph. Among these genes, RNF148 expression significantly increased nearly 1863 times in SW620 as compared to SW48. (**g**) The expression of RNF148 in gastric and colorectal cancer cell lines was determined by RT-PCR. (**h**) Evaluation of RNF148 expression after treatment of 5-FU or BFA. 5-FU, 5-fluorouracil; BFA, Brefeldin A; CHX, Cycloheximide; Leu, leupeptin; CQ, Chloroquine; Ubi, rhHA-ubiquitin

Firstly, we tested the CHAC2 expression in three gastric cancer cell lines and eight colorectal cancer cell lines. *CHAC2* mRNA was expressed in all the tested cell lines, but the protein was hardly detected in most cell lines except SW48 (Figure 1a). Then we quantified the relative mRNA to protein levels in cell lines by real-time PCR (Supplementary Figure S1A). The results revealed that the protein expression levels still correlated with the mRNA expression levels, suggesting that CHAC2 expression was regulated at both translational and post-translational level.

To investigate the biofunction of CHAC2, CHAC2 expression plasmid was transfected into cancer cells to construct stable monoclonal CHAC2-expressed cell lines AGS-CHAC2 and SW620-CHAC2. Meanwhile, control vector plasmid was transfected to construct control cells AGS-vector and SW620-vector. CHAC2 expression in these transfected cell lines was confirmed by RT-PCR, qRT-PCR, and western blot (Figure 1b and Supplementary Figure S1B). We further investigated the effects of 5-fluorouracil (5-FU) and Brefeldin A (BFA) on the expression of CHAC2 in these cells. Western blot results showed that the CHAC2 protein expression was significantly increased after 5-FU or BFA treatment (Figure 1c).

### Ubiquitin-proteasome pathway mediated CHAC2 degradation

Since CHAC2 expression might be regulated at post-translational level, we examined the stability of CHAC2 in AGS-CHAC2 and SW620-CHAC2. It revealed that CHAC2’s half-life (*t*_1/2_) was shorter than 1 h (Figure 1d) by treatment with protein synthesis inhibitor Cycloheximide (CHX). To identify which pathway might involve in CHAC2 degradation, proteasomal (MG-132) or lysosomal (leupeptin (Leu)/chloroquine diphosphate salt (CQ)) inhibitor was used together with CHX. Among them, only MG-132 blocked CHAC2 degradation (Figure 1d). We also observed that the degradation of CHAC2 protein was gradually enhanced with increasing concentration of ubiquitin added to the culture media (Figure 1e). In addition, ERK that mediates serine phosphorylation, ubiquitination and degradation of cortactin^10^ showed correlation with CHAC2 degradation (Figures 1d and e). To explore the specific ubiquitin pathway genes acting on CHAC2, we used the Human Ubiquitination Pathway RT^2^ Profiler PCR Array to compare the expression of 84 key genes involved in the regulated degradation of cellular proteins by the ubiquitin-proteasome between CHAC2 expression silenced SW620 cells and SW48 cells with CHAC2 high expression. The results showed that most genes were upregulated in SW620 cells (Supplementary Table S1). However, what impressed us was that among these genes RNF148 expression significantly increased nearly 1863 times in SW620 as compared to SW48 (Figure 1f). It probably was the specific ubiquitin ligase acting on CHAC2. Then we checked RNF148 expression in all used cell lines, it expressed highly in all CHAC2 nearly silenced cells but not expressed in SW48 (Figure 1g and Supplementary Figure S1C). So its expression was negatively correlated with CHAC2 protein expression. Moreover, we found that 5-FU and BFA treatment could remarkably decrease RNF148 expression (Figure 1h and Supplementary Figure S1D). This is further supported that RNF148 may be the specific ubiquitin ligase acting on CHAC2 since CHAC2 protein expression was significantly increased after 5-FU or BFA treatment. Anyway, more detailed molecular mechanisms of the ubiquitin-proteasome regulation of CHAC2 expression will be studied in the near future.

### Correlation of CHAC2 expression with clinicopathological parameters of patients

**Figure 2:**
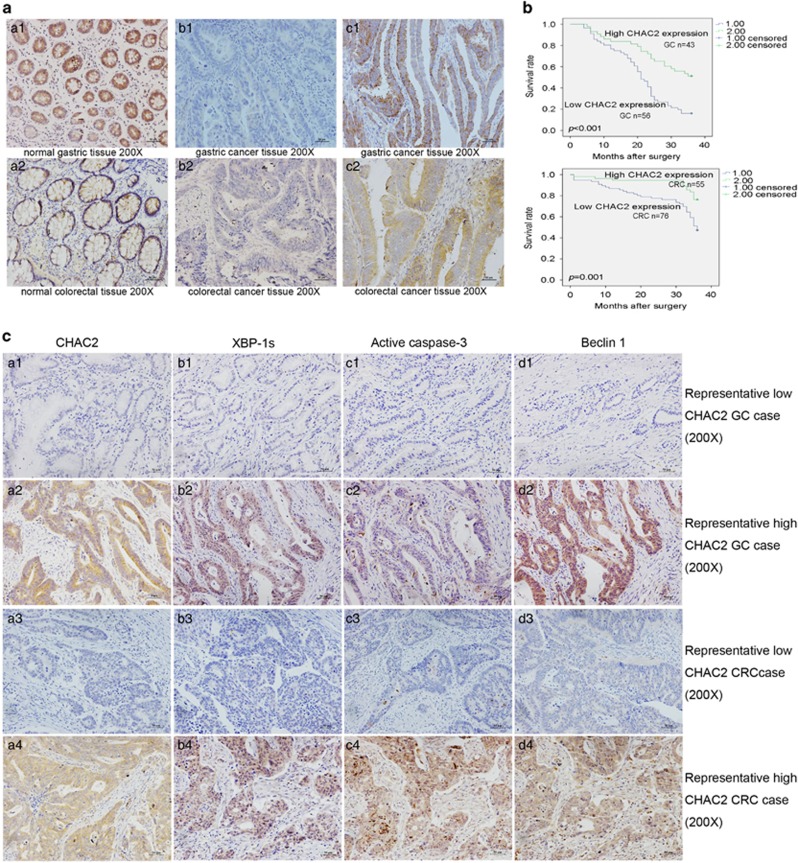
The immunohistochemical results of CHAC2 expression and Kaplan-Meier survival curves. (**a**) Representative immunohistochemical staining for CHAC2 which is mainly expressed in cytoplasm of cells. Original magnification: × 200. (**b**) The overall Kaplan-Meier plots according to CHAC2 expression in patients with gastric and colorectal cancers, which showed a poorer survival in CHAC2-low expression patients than that in CHAC2-high expression patients. The HR for gastric and colorectal cancers were 0.363 (95% CI 0.194–0.678, *P*=0.002) and 0.383 (95% CI 0.227–0.645, *P*<0.001), respectively. (**c**) Representative immunohistochemical staining results showing the positive correlationship between CHAC2 expression and XBP-1s, active caspase-3, or Beclin 1. a1, b1, c1 and d1 is a low CHAC2 expression GC case; a2, b2, c2 and d2 is a high CHAC2 expression GC case; a3, b3, c3 and d3 is a low CHAC2 expression CRC case; a4, b4, c4 and d4 is a high CHAC2 expression CRC case; GC gastric cancer; CRC colorectal cancer. Original magnification: × 200. All the relative quantitive values were put below the correspondent blots

We evaluated the CHAC2 protein expression in primary gastric and colorectal cancer tissues by immunohistochemistry (IHC) and western blot. Representative immunohistochemical staining of CHAC2 protein in normal and cancer tissues was shown in Figure 2a. CHAC2 protein was highly expressed mainly in the cytoplasm of normal tissues. In contrast, CHAC2 was silenced or just low expressed in gastric and colorectal cancer tissues. In addition, representative western blot staining of CHAC2 protein was shown in Supplementary Figure S2. CHAC2 expressed higher in paired non-tumor tissues compared with tumor tissues in 81.25% (13/16) gastric and 87.5% (14/16) colorectal cancer cases, respectively.

The correlations between CHAC2 protein expression and clinicopathological parameters of gastric and colorectal cancer patients were summarized in Supplementary Tables S2 and S3. CHAC2 expression was significantly correlated with histopathological grading (*P*=0.002), depth of invasion (*P*=0.039), lymph node metastasis (*P*=0.017) and TNM stages (*P*=0.011) in gastric cancer patients. In colorectal cancer patients, CHAC2 expression was significantly correlated with lymph node metastasis (*P*<0.001), distant metastasis (*P*=0.017) and TNM stages (*P*<0.001). To further determine the role of CHAC2 expression in gastric and colorectal cancer development, all patients were followed up for overall survival after surgery. Kaplan–Meier survival indicated that the cumulative survival rate of patients with high CHAC2 expression was significantly higher than those with low CHAC2 expression in both gastric (*P*<0.001) and colorectal cancer (*P*=0.001) cases (Figure 2b).

Using univariate analysis, CHAC2 expression (*P*<0.001), the depth of invasion (*P*<0.001), lymph node metastasis (*P*=0.003), distant metastasis (*P*<0.001) and TNM stages (*P*<0.001) were significantly associated with overall survival rates in gastric cancer patients (Supplementary Table S4). In colorectal cancer patients, the CHAC2 expression (*P*=0.002), histopathological grading (*P*=0.013) and distant metastasis (*P*=0.05) were significantly associated with overall survival rates (Supplementary Table S5). Multivariate analysis was further performed depending on the Cox proportional hazards model for all of the significant variables examined in the univariate analysis. We found that the CHAC2 (HR:0.548; 95% CI:0.312–0.960; *P*=0.036) and TNM stages (HR:1.555; 95% CI:1.165–2.076; *P*=0.003) were proved to be independent prognostic factors for survival in gastric cancer (Supplementary Table S6). And the CHAC2 expression (HR:0.380; 95% CI: 0.202–0.711; *P*=0.003) and histopathological grading (HR:1.428; 95% CI: 1.042–1.957; *P*=0.027) were proved to be independent prognostic factors for survival in colorectal cancer (Supplementary Table S7).

### Effects of CHAC2 on the growth and proliferation of cancer cells

**Figure 3:**
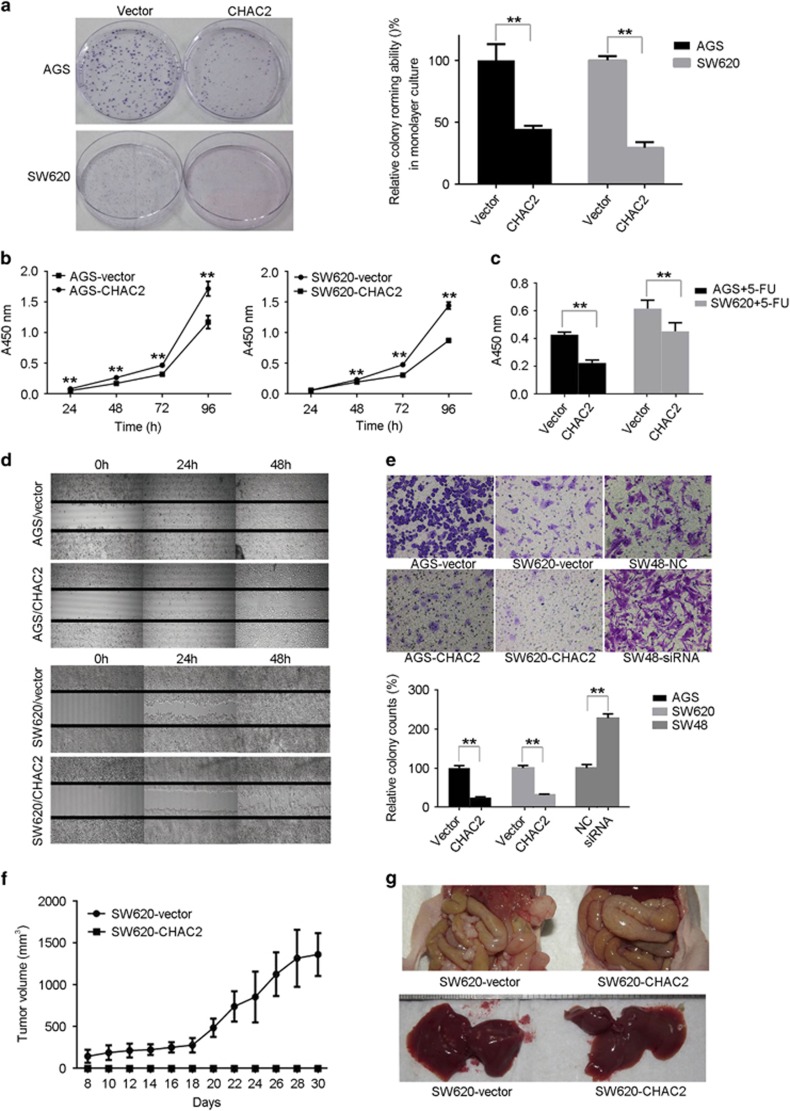
CHAC2 inhibited the growth and migration of gastric and colorectal cancer cells *in vitro* and *in vivo*. (**a**) Representative colony formation assay by monolayer culture and quantitative analysis. The numbers of colonies in each vector transfected control were set to 100%, whereas CHAC2-transfected cells were presented as mean±s.d. (**b**) The cell viability of CHAC2-transfected cells measured without 5-FU treatment. The cell viability was measured every 24 h on a microplate reader at a wavelength of 450 nm. The data was presented as mean±s.d. (**c**) The cell viability of CHAC2-transfected cells was measured with 5-FU treatment. The cell viability with 5-FU was analyzed after 24 h treatment with indicated 5-FU (120 *μ*g/ml for AGS; 200 *μ*g/ml for SW620). The data were presented as mean±s.d. (**d**) Representative results of wound-healing assay. Photos were taken every 24 h, and representatively showed at indicated time (original magnification: × 100). (**e**) Representative results of transwell migration assays and quantitative analysis. The pictures were taken 24 h after seeding (original magnification: × 100). The numbers of migrated cells were counted in five representative high-power fields per transwell. The numbers of colonies in each vector-transfected control were set to 100%, whereas CHAC2-transfected cells were presented as mean±s.d. All the above experiments were carried out in triplicate. Asterisk indicates statistically significant difference (**P<0.01, *P<0.05). (**f**) Effect of CHAC2 expression on tumor growth in nude mice and the quantitative analysis of tumor volume. (**g**) Peritoneal dissemination was observed in the abdominal cavity, mesenterium and liver of all mice inoculated with SW620-vector cells. While no obvious peritoneal dissemination in mice inoculated with SW620-CHAC2 cells

Results above suggested that CHAC2 was a candidate TSG. We investigated the effects of ectopic CHAC2 on the growth of tumor cells. Colony formation assays showed that ectopic CHAC2 expression significantly suppressed the colony formation efficiency of gastric and colorectal cancer cells (Figure 3a, *P*<0.01). Moreover, cell viability assays revealed that ectopic CHAC2 expression significantly suppressed the cell proliferation of gastric and colorectal cancer cells (Figure 3b, *P*<0.01), especially after 5-FU treatment (Figure 3c, *P*<0.01).

### CHAC2 suppressed the migration of gastric and colorectal cancer cells

We investigated the effects of CHAC2 expression on migration of cancer cells by wound-healing assays and transwell migration assays. The results of wound-healing assays showed that the CHAC2-transfected cells took much longer time to close a scratch wound than that of controls (Figure 3d). Similar results were found in transwell migration assays, CHAC2 expression led to significant suppression of gastric and colorectal cancer cell migration. Moreover, CHAC2 knockdown showed promotion effects on wound healing (Supplementary Figure S3) and migration (Figure 3e).

### CHAC2 enhanced apoptosis of cancer cells

**Figure 4:**
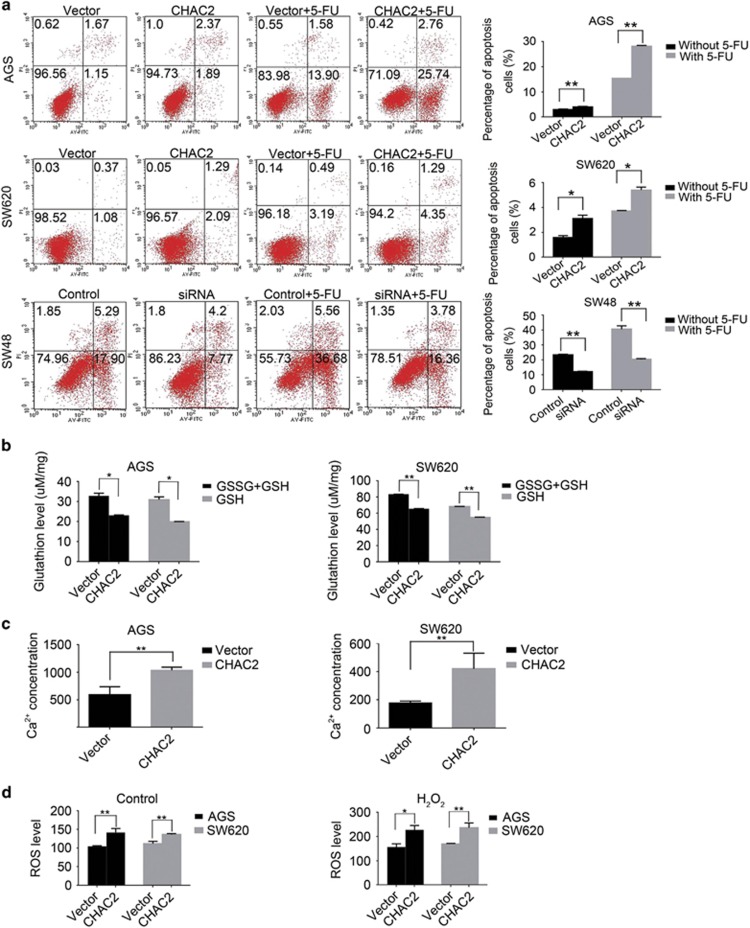
Effect of CHAC2 on apoptosis and production of T-GSH, GSH, Ca^2+^, and ROS of cancer cells. (**a**) Representative results of annexin V-FITC/PI staining and quantitative analysis in CHAC2 overexpression or knockdown cells. Values are mean±s.d of three independent experiments. Asterisk indicates statistically significant difference (**P<0.01, *P<0.05). (**b**) The T-GSH (GSSG+GSH) and GSH concentration of CHAC2-transfected cells comparing with that of controls. (**c**) Effect of CHAC2 on cytoplasmic Ca^2+^ concentration of cancer cells. (**d**) Analysis of ROS production in CHAC2-transfected cells. All the above experiments were performed in triplicate, using vector-transfected cells as control. The data were presented as mean±s.d. Asterisk indicates statistically significant difference (**P<0.01, *P<0.05)

To determine whether tumor cell growth and proliferation inhibition by CHAC2 was related to cell cycle arrest or apoptosis, cell cycle and apoptosis analysis was performed. There was no significant difference in cell cycle phase distribution between CHAC2-transfected cells and controls (Supplementary Figure S4). Regarding cell apoptosis, we checked the spontaneous and 5-FU induced apoptosis of CHAC2-transfected cells and the controls. The percentage of spontaneous and 5-FU induced apoptotic cells was significantly higher in CHAC2-transfected cells relative to the controls (Figure 4a). We further knocked down the CHAC2 expression in SW48 and then checked effects of knockdown on cell apoptosis. The percentage of spontaneous and 5-FU-induced apoptotic cells was significantly lower in CHAC2 knockdown cells as compared with the negative control cells (Figure 4a), which further confirmed that CHAC2 induced tumor cell apoptosis.

### CHAC2 led to glutathione depletion, increased intracellular Ca^2+^ concentration and reactive oxygen species level

Since glutathione depletion is an important factor for apoptosis initiation and execution, the effect of CHAC2 expression on glutathione concentration was evaluated. Both total GSH (T-GSH) and GSH in CHAC2-transfected cells were significantly lower than those of controls (Figure 4b). It was reported that increase of intracellular Ca^2+^ played a central role in cell death,^11^ we subsequently investigated the effects of CHAC2 expression on intracellular Ca^2+^ concentration. It revealed that CHAC2-transfected cells showed higher intracellular Ca^2+^ concentration than that of controls (Figure 4c). Mitochondrial Ca^2+^ handling regulates several important processes in cellular physiology including mitochondrial reactive oxygen species (ROS) generation.^12^ Similar to the alteration of intracellular Ca^2+^ concentration, CHAC2-transfected cells showed increased ROS level comparing with that of controls (Figure 4d).

### CHAC2 induces UPR

**Figure 5:**
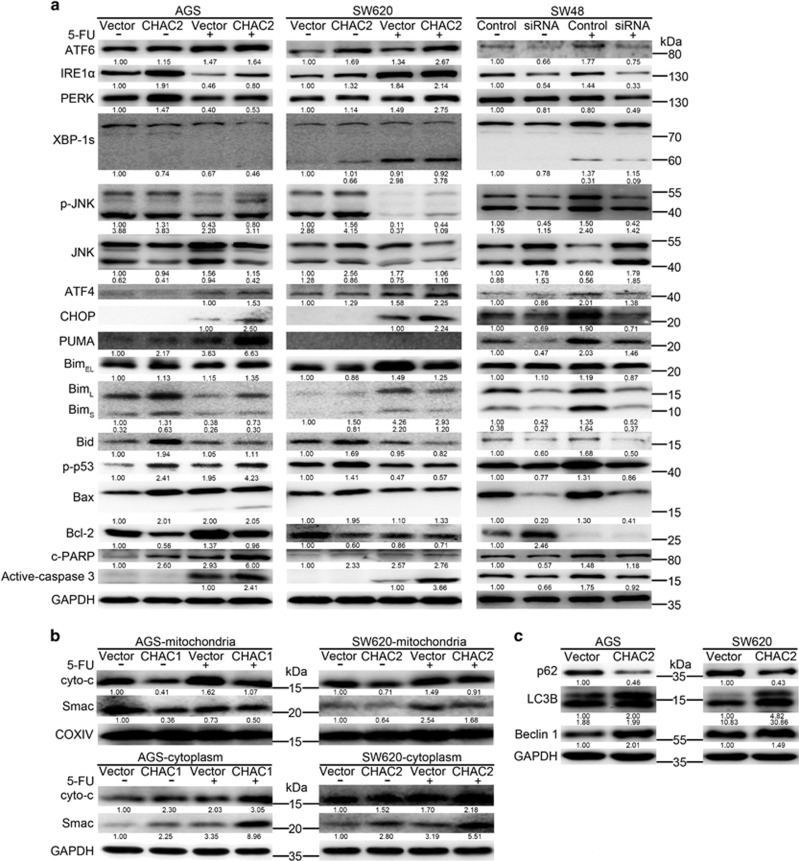
Effects of CHAC2 on the cancer cell apoptosis, autophagy and their related proteins. (**a**) Expression of several key UPR regulators and cell apoptotic regulators examined by western blot in CHAC2 overexpression or knockdown cells. (**b**) Expression levels of apoptosis factor cyto c and Smac in mitochondria and cytoplasmic of cancer cells were evaluated in CHAC2-transfected cells, using vector transfected cells as control. (**c**) Determination of the expression of key autophagic regulators by western blot. 5-FU, 5-fluorouracil; c-PARP, cleaved PARP; cyto c, cytochrome c. All the relative quantitive values were put below the correspondent blots

CHAC2 led to GSH depletion, which would disturb the oxidation reduction potential of cell and then resulted in triggering UPR. We subsequently investigated the correlation of CHAC2 expression with UPR by western blot. All the three ER resident stress sensors including PERK, IRE1 and ATF6 were significantly upregulated in CHAC2-transfected cells exposed with or without 5-FU. After treatment with or without 5-FU, the important components of UPR, including ATF4, CHOP and XBP-1s, were upregulated in CHAC2-transfected cells than those of controls (Figure 5a). On the contrary, in SW48 cells, these important components of UPR were downregulated in CHAC2 knockdown cells as compared with the negative control cells (Figure 5a).

### CHAC2 promoted mitochondrial apoptosis

In response to ER stress, the intrinsic pathway is triggered, where mitochondria plays a central role in the control of apoptosis.^13^ The collapse of the mitochondrial transmembrane potential led to releasing of mitochondrial apoptogenic factor cytochrome c (cyto c) and Smac, so the cytosolic and mitochondrial fractions were analyzed by immunoblotting. Moreover, most stimuli that induce mitochondria-dependent apoptosis is controlled by proteins of the Bcl-2 family, so we checked pro-apoptotic proteins PUMA, Bim, Bid, p-p53, Bax and pro-survival members Bcl-2 by western blot. Our results showed that lots of pro-apoptotic proteins were upregulated in CHAC2-transfected cells (Figure 5a). However, in SW48 cells, the pro-apoptotic proteins were downregulated in CHAC2 knockdown cells than that of the negative control cells (Figure 5a). Meanwhile, anti-apoptotic protein Bcl-2 was decreased in CHAC2-transfected cells than that of controls, and was upregulated in CHAC2 knockdown SW48 cells as compared with the negative control cells (Figure 5a). Further detection showed that mitochondrial cyto c of CHAC2-transfected cells was decreased, while its cytoplasmic cyto c was increased as predicted. Similar results were observed regarding to Smac (Figure 5b).

### Effect of CHAC2 on autophagy of gastric and colorectal cancer cells

Autophagy is widely involved in the pathogenesis of many diseases, especially cancers.^14^ Current evidence supports the idea that autophagic cell death suppresses tumorigenesis.^15^ We evaluated the effect of CHAC2 expression on cells which were transiently transfected with GFP-LC3 plasmids. More punctate fluorescence was observed in CHAC2-transfected cells than that of controls, along with increased autophagy LC3 II and decreased p62 (Figure 5c). These results suggested that CHAC2 expression contributed to autophagy induction in gastric and colorectal cancer cells.

### CHAC2 expression was positively correlated with XBP-1s, active caspase-3, or Beclin 1 in clinical samples

As described above, *in vitro* study CHAC2 could induce mitochondrial apoptosis and autophagy through UPR. We selected XBP-1s, active caspase-3 and Beclin 1 as the protein markers of UPR, apoptosis, and autophagy, respectively. IHC was performed to determine the correlationship between CHAC2 and these three protein markers in clinical samples. In gastric cancer, high CHAC2 expression was correlated with XBP-1s, active caspase-3 or Beclin 1 high expression with a correlation coefficient of 0.305 (*P*=0.002), 0.607 (*P*<0.001) and 0.591 (*P*<0.001), respectively (Supplementary Table S8). In colorectal cancer, high CHAC2 expression was also correlated with XBP-1s, active caspase-3 or Beclin 1 high expression with a correlation coefficient of 0.526 (*P*<0.001), 0.319 (*P*<0.001) and 0.433 (*P*<0.001), respectively (Supplementary Table S8). Representative IHC results are shown in Figure 2c. Therefore, *in vivo* study also showed that CHAC2 could induce mitochondrial apoptosis and autophagy through UPR.

### CHAC2 inhibited tumor growth and peritoneal metastasis in nude mice

At last, we evaluated the effects of CHAC2 on tumor cell growth *in vivo* by injection of SW620-CHAC2 and SW620-vector into BALB/c nude mice cells (*n*=6, respectively). The growth curve of CHAC2-transfected and vector-transfected SW620 cells in nude mice is shown in Figure 3f. No obvious tumor was observed from the BALB/c nude mice injected with CHAC2-transfected cells. We further investigated whether CHAC2 expression had a suppressive effect on peritoneal metastasis by *in vivo* model. Peritoneal dissemination visualized as innumerable whitish nodules was observed in the abdominal cavity, mesenterium and liver of all mice inoculated with SW620-vector cells. While no obvious peritoneal dissemination in mice inoculated with SW620-CHAC2 cells (Figure 3g).

## Discussion

In this study, we checked the expression of CHAC2 in gastric and colorectal cancer cell lines and primary tissues. All the tested cell lines exhibited CHAC2 mRNA expression, but only SW48 showed obvious CHAC2 protein expression, suggesting that the CHAC2 expression might be regulated at the level of translation or post translation. Though the quantified results of the relative mRNA to protein levels suggested that CHAC2 expression was regulated at both translational and post-translational level. We found CHAC2 half-life was shorter than 1 h. The ubiquitin-proteasome pathway but not the lysosome pathway was mainly involved in CHAC2 degradation. Similar result was once reported for mouse CHAC1 where CHAC1 protein expression was only detected in the presence of proteasome inhibitor (MG-132) though its mechanism is still unknown.^16^ In addition, ERK, which mediates serine phosphorylation, ubiquitination and degradation of cortactin, was also involved in CHAC2.^10^ Our study results have pointed out that RNF148 may be the specific ubiquitin ligase acting on CHAC2. RNF148 was just identified as a novel gene recently.^17^ It is abundantly expressed in testis and slightly expressed in pancreas but not in other tissues including normal gastric and colorectal mucosa. RNF148 encodes a polypeptide of 305 amino acids (AA), including a C3H2C3- type RING-H2 finger domain at the C-terminus from AA residues 258 to 299. This domain has a high identity with that of other human E3 ubiquitin ligases such as RNF128,^18^ RNF130,^19^ and mouse Rnf133.^20^ *In vitro* ubiquitination reaction has shown that the recombinant RNF148 protein has E3 ubiquitin ligase activity.^21^ Now we found for the first time that RNF148 may have an important role in human gastric and colorectal cancer development through its E3 ubiquitin ligase function though more detailed molecular mechanisms of its ubiquitin-proteasome regulation of CHAC2 expression will be studied further.

In both gastric and colorectal cancer cases, CHAC2 was found to be frequently downregulated. CHAC2 expression was significantly correlated with many clinicopathological parameters of patients. These findings, together with the results that restoration CHAC2 expression inhibited tumor cell growth, proliferation and migration *in vitro* and *in vivo*, suggested that CHAC2 acted as a tumor suppressor in gastric and colorectal cancers. Sequence alignment of CHAC2 homologues revealed that it shares 50% AA identity with CHAC1. Similar to CHAC1, CHAC2 also contains signature motif and catalytic glutamate residue and belongs to the family of *γ*-GCTs (Supplementary Figure S5). We showed that CHAC2 led to depletion of T-GSH and GSH. GSH acts as a major antioxidant within cells by maintaining a tight control of the redox status.^22^ GSH depletion disturbing the oxidation reduction potential of cell will result in ER stress and triggering UPR. In this study, all the three major ER stress sensors: IRE1, ATF6 and PERK, together with XBP-1 s and ATF4 were upregulated in CHAC2-transfected cells. Each sensor uses a unique mechanism to activate and regulate ER stress target genes.^23^ Among them, IRE1*α* excises the mRNA of XBP-1s to generate spliced XBP-1 (XBP-1s) which is translocated to the nucleus to induce the upregulation of its target genes.^24, 25, 26, 27, 28^ Under ER stress conditions, ATF6 translocates to the Golgi, following releasing a cytosolic fragment that directly controls genes encoding ERAD components and XBP-1s. Finally,^29, 30^ PERK phosphorylates eIF2*α*. eIF2*α* selective translate of the transcription factor ATF4.^31^

**Figure 6:**
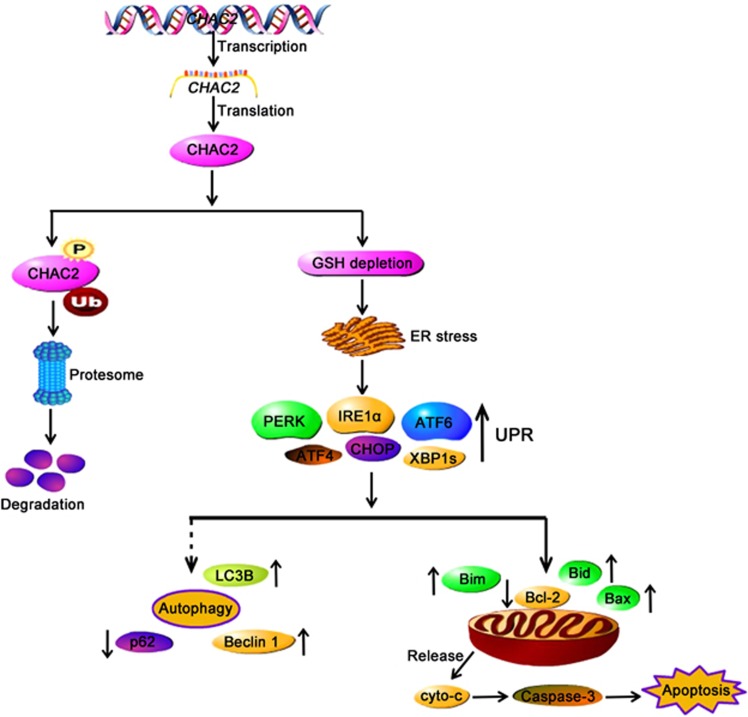
Diagram showing the mechanisms of CHAC2. The downregulated CHAC2 can be degraded by ubiquitin-proteasome pathway in gastric and colorectal cancers. CHAC2 induces UPR and promotes the mitochondrial apoptotic pathway by releasing cyto c which further activates caspase-dependent signaling. ER, endoplasmic reticulum; UPR, unfolded protein response

Unmitigated ER stress induces apoptosis to eliminate irreversibly damaged cells. Cell death under ER stress depends on the core mitochondrial apoptotic pathway.^32^ It is reported that sustained PERK signaling makes ATF4 upregulating the pro-apoptotic transcription factor CHOP. CHOP upregulates the transcription of Bim and downregulates the anti-apoptotic protein Bcl-2 which is crucial for the control of ER stress-induced apoptosis.^33, 34^ In CHAC2-transfected cells, we observed that CHOP, Bax, and Bak were upregulated, while Bcl-2 was dowregulated. CHOP can also upregulate BH3-only proteins which regulate the activation of Bax or Bak to trigger apoptosis. In addition, Bax or Bak can be also upregulated by IRE1*α*-JNK and ATF6-Bid, which were upregulated in CHAC2-transfected cells. Meanwhile, CHOP also upregulates growth arrest and DNA damage-inducible34 (GADD34) which may induce the generation of ROS. This was further confirmed by the fact that the level of ROS was increased in CHAC2-transfected cells than that of controls. In addition to ROS, altered calcium homeostasis, which was observed in CHAC2-transfected cells, may also contribute to the opening of the mitochondrial permeability transition pore (PTP) and promote apoptosis. Further detection showed that both cyto c and Smac were decreased in mitochondria and increased in cytoplasm, suggesting that opening of the mitochondrial PTP makes the mitochondrial cyto c and Smac releasing to cytoplasm. Release of cyto c leads to caspase-3 activation, which was upregulated in CHAC2-transfected cells, and subsequent apoptosis. Our findings showed that CHAC2 induced UPR and stimulated the mitochondrial apoptotic pathway by releasing cyto c that further activates caspase-dependent signaling (Figure 6).

Our results showed that CHAC2 induced mitochondrial apoptosis and autophagy through UPR. Both apoptosis and autophagy play an important role in the cell death, normal physiology, and cellular homeostasis, and disturbance of these regulatory pathway may lead to different human diseases including cancers.^14, 35^ Autophagy plays dual roles during cell life; on the one hand, it facilitates cell survival under stressful conditions, on the other hand, excessive or persistent autophagy promotes cell death. Enhanced UPR and autophagy were observed in CHAC2-transfected cells. It is reported that UPR attenuate protein synthesis at the ER by inhibiting translation, activating mRNA decay, and activating autophagy through the IRE1*α*-JNK or PERK-ATF4 pathway.^36, 37^ Our study showed that all these proteins, IRE1*α*, JNK, PERK and ATF4, were upregulated in CHAC2-transfected cells. Accumulating evidences suggest that the effects of anticancer therapies are not confined to apoptosis but also involve autophagy. The crosstalk between autophagy and apoptosis is quite complicated; on the one hand, some common upstream signals result in the activation of combined autophagy and apoptosis, on the other hand, they may be also mutually exclusive under certain conditions.^38^ Further studies are needed to explore the mechanisms of crosstalk between apoptosis and autophagy mediated by CHAC2.

In conclusion, this study provides the first evidence that CHAC2 was downregulated and degraded by ubiquitin-proteasome pathway in gastric and colorectal cancers. And CHAC2 acted as a tumor suppressor inducing mitochondrial apoptosis and autophagy simultaneously through UPR. A better understanding of the molecular mechanisms of CHAC2 in gastrointestinal cancer development may lead to a more effective management of gastric and colorectal cancer patients with the low expression of CHAC2.

## Materials and methods

### Cell lines, tumor and normal control samples

Three gastric cancer (MKN28, MKN45, and AGS) and eight colorectal cancer cell lines (SW620, SW480, SW48, RKO, HT29, HCT116, HCT8, and DLD1) were used. The cell lines were maintained at 37 °C in a humidified 5% CO_2_ incubator in RPMI 1640 or Dulbecco’s modified Eagle’s medium (Gibco BRL, Rockville, MD, USA) supplemented with 10% fetal bovine serum (FBS).

For the IHC experiments, 10 normal gastric and 10 normal colonic mucosa biopsy samples were used as normal controls. A total of 99 gastric and 131 colorectal cancer patients who underwent surgery between February 2004 and June 2006 at the Sir Run Run Shaw Hospital (Hangzhou, Zhejiang, China) were investigated. All these patients included in this study had not received preoperative radiotherapy, chemotherapy or immunotherapy before surgery.

### RNA extraction and semi-quantitative RT-PCR

Total RNA was extracted using the Trizol reagent (Invitrogen, Carlsbad, CA, USA) as described by the manufacturer. The mRNA expression levels of the CHAC2 were determined by semi-quantitative reverse-transcription PCR (RT-PCR) with GoTaq polymerase (Promega, Madison, WI, USA). The transcription of the principal gene GAPDH was used as the internal control. Specific primers were designed according to CHAC2 sequence (CHAC2F: 5′-ATGTGGGTTTTTGGTTACGG-3′ CHAC2R: 5′-TTGTTGTGGGATCTTTTGGA-3′).

### Protein extraction and western blot

Cells were collected from cultured dishes and were lysed in a RIPA lysis buffer (Beyotime, Hangzhou, Zhejiang, China) supplemented with inhibitors of proteases. Protein concentration was determined using a BCA Protein Assay Kit (Beyotime). Cell lysates (40 *μ*g protein/line) were separated on a 6–15% SDS-PAGE and transferred to nitrocellulose membrane (Bio-Rad, Hercules, CA, USA). The blotted membranes were blocked in 5% skim milk for 1 h and incubated over-night at 4 °C with the following primary antibodies (1:1000). Afterward, the blotted membranes were incubated with HRP-labeled secondary antibody (1:2000) in TBST for 1 h. Primary and secondary antibodies used were purchased from Cell Signaling Technologies (Danvers, MA, USA). Detection was carried out using ECL Kit (Pierce Chemical Co., Rockford, IL, USA) and the blots were developed using a Fujifilm Las-4000 Imaging System.

### Immunohistochemistry

IHC was performed using the ChemMate EnVision detection kit (Dako, Carpinteria, CA, USA) as described by the manufacturer. Briefly, the selected sections were incubated with primary CHAC2 antibody (HPA049235, Atlas Antibodies AB, Stockholm, Sweden), XBP-1s (647502, BioLegend, San Diego, CA, USA), active caspase-3 (BS7004, Bioworld, Minneapolis, MN, USA), or Benclin 1 (sc-48341, Santa Cruz, Santa Cruz, CA, USA) overnight at 4 °C, and then incubated with ChemMate EnVision/HRP, Rabbit/Mouse reagent as a secondary antibody. Afterwards, the sections were developed using ChemMate DAB+ chromogen and counterstained with hematoxylin.

Two independent investigators, who were blinded to patient-related clinical information, evaluated slides on staining intensity, subcellular localization, and the percentage of positive cells three times. To evaluate the association of CHAC2 expression with clinical and pathological parameters, the patients were then grouped into two categories, low-expression and high-expression, based on the percentage of positive cells and the intensity of stained cells.

### Construction of stable CHAC2-expressing cell lines

To construct stable CHAC2-expressing cell lines, MegaTran 1.0 transfection reagent (Origene, Rockville, MD, USA) was used according to its instruction. Gastric cancer cell line AGS and colorectal cancer cell line SW620 were transfected with pCMV6 entry-CHAC2 plasmid (pCMV6 entry-CHAC2; Origene) or control vector (pCMV6 entry-mock; Origene). Stable CHAC2-expressing and control clones were selected for further study.

### RNA interference of CHAC2

siRNA sequence for CHAC2 and the control siRNA were designed using Ambion software, and were synthesized and purchased from GenePharma, Shanghai, China (CHAC2 siRNA: Sense 5′-GAAGACAUUGCUGAACAAATT-3′, Antisense 5′-UUUGUUCAGCAAUGUCUUCTT-3′ Negative control siRNA: Sense 5′-UUCUCCGAACGUGUCACGUTT-3′, Antisense 5′-ACGUGACACGUUCGGAGAATT-3′). siRNA transfection was performed with Lipofectamine RNAi Max transfection reagent (Invitrogen) according to the manufacturer’s instructions. Seventy-two hours later, the total protein of SW48 cells in each group was extracted and the efficiency of siRNA was confirmed by western blot.

### Colony formation assays

For colony formation assays, 1000 stable transfected cells were plated in a 10 cm dish and allowed to grow for 2 weeks at 37 °C in 5% CO_2_. Surviving colonies (⩾ 50 cells/colony) were counted under a microscope after Giemsa staining. The experiments were performed in triplicate.

### Cell viability assay

Cell viability assay was performed using the CCK8 detection kit (Dojindo, Kumamoto, Japan) as described by the manufacturer. Briefly, cells were seeded in 96-well flat-bottomed microtitre plates at a density of 5000 cells/well. After 24 h, the medium was replaced by medium without or with indicated 5-fluorouracil (5-FU; 120 *μ*g/ml for AGS; 200 *μ*g/ml for SW620) and incubated for 24 h. Absorbance was measured on a microplate reader (Synergy H4 Hybrid Reader, Bio-Tek, Winooski, VT, USA) at a wavelength of 450 nm. The experiments were performed in triplicate.

### Wound-healing assay

Cell motility was assessed using a scratch wound assay. The transfected cells and the controls were cultured in 6-well dishes with 10 μg/l mitomycin C (MCE, Princeton, NJ, USA) until confluent. The cell layers were carefully wounded using sterile tips and washed twice with fresh medium. Cells were incubated with fresh medium and photographed under a phase contrast microscope at different hours after wounding. The experiments were performed in triplicate.

### Cell migration assay

For the transwell assay, cells were trypsinized and resuspended in corresponding medium containing 1% FBS at a density of 1 × 10^6^ cells/ml with 10 μg/l mitomycin C. One hundred microliters of the cell suspension was added into the upper chamber of a transwell (Corning, Corning, NY, USA) consisted of inserts containing 8-mm pore-size PET membrane. Six hundred microliters medium containing 10% FBS was placed in the lower chamber. After indicated hours incubation at 37 °C in 5% CO_2_, cells remained in the upper chamber was removed carefully by cotton swab, and cells on the bottom side of the chamber membrane were fixed, stained with 0.25% crystal violet, photographed and counted under a light microscope. The experiments were performed in triplicate.

### Cell cycle and apoptosis analysis

Cell cycle distribution and the percentage of apoptosis were determined by flow cytometry according to the manufacturer’s instruction (BD, San Jose, CA, USA).

### Measurement of total GSH, GSH and GSSG concentration

The protein concentration of treated cells was determined using a BCA Protein Assay Kit (Beyotime). The concentrations of reduced glutathione (GSH) and total GSH (T-GSH) were measured using GSSG Assay Kit (Beyotime) as described by the manufacturer.^39^ Briefly, T-GSH was assayed using the 5,5-dithio-bis (2-nitrobenzoic)acid (DTNB)-GSSG reductase recycling. GSSG was measured by measuring 5-thio-2-nitrobenzoic acid (TNB) which was produced from the reaction of reduced GSH with DTNB. The concentration of reduced GSH in the sample was obtained by subtracting GSSG from T-GSH.

### Measurement of Ca^2+^ concentration

Measurement of Ca^2+^ concentration was performed using the Fluo-3 AM (Beyotime) according to the protocol with little modification.^40^ Calibrate and express the signals as [Ca^2+^]_*i*_ using a pseudo calibration method, as described by Trafford *et al.*^41^ using the equation: [Ca^2+^]_*i*_=*K*_d_(*F*−*F*_bg_)/(*F*_max_−*F*), where *F* is the fluorescence signal, *F*_bg_ is the fluorescence in the absence of Ca^2+^, *F*_max_ is the fluorescence in the presence of saturating Ca^2+^, and *K*_d_ (400 nM/l) the dissociation constant of Fluo-3AM.

### Measurement of reactive oxygen species production

C-H_2_DCFDA (Invitrogen) was used to measure ROS production as described by the manufacturer. Briefly, 5 × 10^5^ cells were seeded into six-well plate, indicated H_2_O_2_ was added into the culture medium when cells were 70% confluent. After 24 h incubation, treated cells were stained with 8 *μ*M C-H_2_DCFDA for 20 min, and washed thrice in PBS before flow cytometry analysis.

### Autophagy detection

Autophagosome formation is one of the features of autophagy and can be detected by endogenous LC3 or GFP-LC3 puncta incorporating into autophagic vacuoles. Autophagy detection was determined as described previously.^42^ Briefly, cells were seeded on 12 mm cover slips into six-well at a density of 5 × 10^5^ cel|ls/well. After attachment overnight, the cells were transiently transfected with a GFP-LC3-expressing vector (Origene). After 32 h of treatment, the cells were fixed with 4% formaldehyde, then observed and photographed under fluorescent microscopy. Cells representing several intense punctate GFP-LC3B aggregates with no nuclear GFP-LC3B were classified as autophagic.

### The human ubiquitination pathway RT^2^ profiler PCR array

The Human Ubiquitination Pathway RT^2^ Profiler PCR Array (Cat. no. 330231, Qiagen, Hilden, German) was used to profile the expression of 84 key genes involved in the regulated degradation of cellular proteins by the ubiquitin-proteasome. Total RNA was extracted from SW620 and SW48 with TRIzol. Single stranded cDNA was synthesized from 2 *μ*g of total RNA by using the SuperArray reaction ready first strand cDNA synthesis kit. The cDNAs were mixed with SuperArray RT^2^ Real time SYBR Green/ROX PCR master mix and real-time PCR performed in accordance with the manufacturer’s instructions. Thermal cycling and fluorescence detection were performed using an ABI ViiA^TM^ 7 Dx System (Applied Biosystems, Foster City, CA, USA), and expression of these 84 E3 ubiquiting ligases gene transcripts was compared between SW620 and SW48.

### *In vivo* subcutaneous tumor model

All of the *in vivo* experimental protocols were approved by the animal care committee of Sir Run Run Shaw Hospital, Zhejiang University. Viable CHAC2-transfected cells and their controls (5 × 10^6^ cells in 0.1 ml PBS) were injected subcutaneously into left forelimb flank of 5-week-old female BALB/c nude mice (six mice per group). Tumor volume was assessed every 2 days for 4 weeks. Tumor volume was calculated by the following formula: (short diameter)^2^ × (long diameter)/2.

### *In vivo* peritoneal metastasis model

Viable cells (1 × 10^7^ cells in 0.5 ml PBS) were injected into the peritoneal cavity of 5-week-old female BALB/c nude mice (six mice per group). All mice were killed after 15 days. We determined the number of tumor nodules and the presence or absence of any bloody ascites as described before.^43^

### Statistical analysis

Results are expressed as values of mean±standard deviation (s.d.). Statistical analysis was carried out using the SPSS 22.0 software package (SPSS, IBM, Chicago, IL, USA). Pearson’s Chi-squared tests and Fisher’s exact test were used to analyze the association of CHAC2 expression with clinicopathological parameters. The Kaplan–Meier method was used to analyze survival curve, and the differences were conducted by the log-rank test. Cox’s proportional hazards regression model was utilized for univariate and multivariate analyses. We performed paired *t*-test (two-tailed) statistical analysis. *P*<0.05 was considered statistically significance.

## Publisher’s Note

Springer Nature remains neutral with regard to jurisdictional claims in published maps and institutional affiliations.
