# Supplementary material for: CHAC2, downregulated in gastric and colorectal cancers, acted as a tumor suppressor inducing apoptosis and autophagy through unfolded protein response
Source: Cell Death Dis. 2017 Aug 24;8(8):e3009–. doi: 10.1038/cddis.2017.405 (PMC5596586; doi:10.1038/cddis.2017.405)
Supplement: Supplementary Tables [file cddis2017405x1.doc]

**Supplementary tables**

**Table S1.** The relative expression of ubiquitylation pathway proteins detected by qRT-PCR in SW620 VS SW48

| **Functional group** | **Gene name** | **Refseq** | **Description** | **Fold expression** |
| --- | --- | --- | --- | --- |
| Ubiquitin-activating enzymes (E1) | ATG7 | NM_006395 | ATG7 autophagy related 7 homolog (S. cerevisiae) | 1.101 |
|  | MOCS3 | NM_014484 | Molybdenum cofactor synthesis 3 | 1.572 |
|  | NAE1 | NM_003905 | NEDD8 activating enzyme E1 subunit 1 | 9.048 |
|  | NEDD8 | NM_006156 | Neural precursor cell expressed, developmentally down-regulated 8 | 1.300 |
|  | SAE1 | NM_005500 | SUMO1 activating enzyme subunit 1 | 3.096 |
|  | UBA1 | NM_003334 | Ubiquitin-like modifier activating enzyme 1 | 2.402 |
|  | UBA2 | NM_005499 | Ubiquitin-like modifier activating enzyme 2 | 4.844 |
|  | UBA3 | NM_003968 | Ubiquitin-like modifier activating enzyme 3 | 1.832 |
|  | UBA5 | NM_198329 | Ubiquitin-like modifier activating enzyme 5 | 2.272 |
|  | UBA6 | NM_018227 | Ubiquitin-like modifier activating enzyme 6 | 3.556 |
|  | UBE2Z | NM_023079 | Ubiquitin-conjugating enzyme E2Z | 1.206 |
| Ubiquitin-conjugating enzymes (E2) | ANAPC2 | NM_013366 | Anaphase promoting complex subunit 2 | 0.246 |
|  | ARIH1 | NM_005744 | Ariadne homolog, ubiquitin-conjugating enzyme E2 binding protein, 1 (Drosophila) | 1.835 |
|  | BARD1 | NM_000465 | BRCA1 associated RING domain 1 | 1.989 |
|  | BRCA1 | NM_007294 | Breast cancer 1, early onset | 2.829 |
|  | BTRC | NM_033637 | Beta-transducin repeat containing | 4.679 |
|  | CDC34 | NM_004359 | Cell division cycle 34 homolog (S. cerevisiae) | 1.226 |
|  | PARK2 | NM_004562 | Parkinson protein 2, E3 ubiquitin protein ligase (parkin) | 0.135 |
|  | STUB1 | NM_005861 | STIP1 homology and U-box containing protein 1, E3 ubiquitin protein ligase | 1.128 |
|  | TMEM189 | NM_199129 | Transmembrane protein 189 | 3.185 |
|  | TP53 | NM_000546 | Tumor protein p53 | 4.251 |
|  | UBE2A | NM_003336 | Ubiquitin-conjugating enzyme E2A | 4.138 |
|  | UBE2B | NM_003337 | Ubiquitin-conjugating enzyme E2B | 2.133 |
|  | UBE2C | NM_181803 | Ubiquitin-conjugating enzyme E2C | 5.840 |
|  | UBE2D1 | NM_003338 | Ubiquitin-conjugating enzyme E2D 1 | 2.232 |
|  | UBE2D2 | NM_181838 | Ubiquitin-conjugating enzyme E2D 2 | 3.499 |
|  | UBE2D3 | NM_181893 | Ubiquitin-conjugating enzyme E2D 3 | 1.937 |
|  | UBE2E1 | NM_182666 | Ubiquitin-conjugating enzyme E2E 1 | 2.909 |
|  | UBE2E2 | NM_152653 | Ubiquitin-conjugating enzyme E2E 2 | 1.550 |
|  | UBE2E3 | NM_006357 | Ubiquitin-conjugating enzyme E2E 3 | 3.761 |
|  | UBE2G1 | NM_003342 | Ubiquitin-conjugating enzyme E2G 1 | 4.039 |
|  | UBE2G2 | NM_182688 | Ubiquitin-conjugating enzyme E2G 2 | 2.750 |
|  | UBE2H | NM_182697 | Ubiquitin-conjugating enzyme E2H | 0.363 |
|  | UBE2I | NM_003345 | Ubiquitin-conjugating enzyme E2I | 2.768 |
|  | UBE2J1 | NM_016021 | Ubiquitin-conjugating enzyme E2, J1, U | 3.931 |
|  | UBE2J2 | NM_194458 | Ubiquitin-conjugating enzyme E2, J2 | 2.212 |
|  | UBE2K | NM_005339 | Ubiquitin-conjugating enzyme E2K | 1.953 |
|  | UBE2L3 | NM_003347 | Ubiquitin-conjugating enzyme E2L 3 | 2.849 |
|  | UBE2M | NM_003969 | Ubiquitin-conjugating enzyme E2M | 1.192 |
|  | UBE2N | NM_003348 | Ubiquitin-conjugating enzyme E2N | 3.497 |
|  | UBE2Q1 | NM_017582 | Ubiquitin-conjugating enzyme E2Q family member 1 | 1.330 |
|  | UBE2R2 | NM_017811 | Ubiquitin-conjugating enzyme E2R 2 | 1.829 |
|  | UBE2S | NM_014501 | Ubiquitin-conjugating enzyme E2S | 1.833 |
|  | UBE2T | NM_014176 | Ubiquitin-conjugating enzyme E2T (putative) | 1.936 |
|  | UBE2W | NM_018299 | Ubiquitin-conjugating enzyme E2W (putative) | 1.792 |
|  | UBR2 | NM_015255 | Ubiquitin protein ligase E3 component n-recognin 2 | 2.931 |
| Ubiquitin-protein ligases (E3) | ANAPC11 | NM_001002244 | Anaphase promoting complex subunit 11 | 2.887 |
|  | BRCC3 | NM_024332 | BRCA1/BRCA2-containing complex, subunit 3 | 1.346 |
|  | CBL | NM_005188 | Cas-Br-M (murine) ecotropic retroviral transforming sequence | 1.340 |
|  | CUL1 | NM_003592 | Cullin 1 | 3.344 |
|  | CUL2 | NM_003591 | Cullin 2 | 4.044 |
|  | CUL3 | NM_003590 | Cullin 3 | 3.622 |
|  | CUL4A | NM_003589 | Cullin 4A | 4.375 |
|  | CUL4B | NM_003588 | Cullin 4B | 3.773 |
|  | CUL5 | NM_003478 | Cullin 5 | 4.482 |
|  | CUL7 | NM_014780 | Cullin 7 | 0.608 |
|  | CUL9 | NM_015089 | Cullin 9 | 0.742 |
|  | DDB1 | NM_001923 | Damage-specific DNA binding protein 1, 127kDa | 2.533 |
|  | DZIP3 | NM_014648 | DAZ interacting protein 3, zinc finger | 1.607 |
|  | FBXO3 | NM_012175 | F-box protein 3 | 3.070 |
|  | FBXO31 | NM_024735 | F-box protein 31 | 1.084 |
|  | FBXO4 | NM_012176 | F-box protein 4 | 1.260 |
|  | FBXW10 | NM_031456 | F-box and WD repeat domain containing 10 | 0.124 |
|  | FBXW9 | NM_032301 | F-box and WD repeat domain containing 9 | 2.028 |
|  | HECW1 | NM_015052 | HECT, C2 and WW domain containing E3 ubiquitin protein ligase 1 | 0.078 |
|  | HECW2 | NM_020760 | HECT, C2 and WW domain containing E3 ubiquitin protein ligase 2 | 0.028 |
|  | HERC5 | NM_016323 | Hect domain and RLD 5 | 0.001 |
|  | HUWE1 | NM_031407 | HECT, UBA and WWE domain containing 1 | 1.657 |
|  | 5-Mar | NM_017824 | Membrane-associated ring finger (C3HC4) 5 | 1.110 |
|  | MDM2 | NM_002392 | Mdm2 p53 binding protein homolog (mouse) | 1.055 |
|  | MIB1 | NM_020774 | Mindbomb homolog 1 (Drosophila) | 4.269 |
|  | MUL1 | NM_024544 | Mitochondrial E3 ubiquitin protein ligase 1 | 0.737 |
|  | RFWD2 | NM_022457 | Ring finger and WD repeat domain 2 | 1.754 |
|  | RNF123 | NM_022064 | Ring finger protein 123 | 1.040 |
|  | RNF148 | NM_198085 | Ring finger protein 148 | 1863.270 |
|  | SKP1 | NM_006930 | S-phase kinase-associated protein 1 | 2.459 |
|  | SKP2 | NM_005983 | S-phase kinase-associated protein 2 (p45) | 1.044 |
|  | SMURF1 | NM_020429 | SMAD specific E3 ubiquitin protein ligase 1 | 1.934 |
|  | SMURF2 | NM_022739 | SMAD specific E3 ubiquitin protein ligase 2 | 2.946 |
|  | SYVN1 | NM_172230 | Synovial apoptosis inhibitor 1, synoviolin | 1.861 |
|  | UBE4B | NM_006048 | Ubiquitination factor E4B | 2.964 |
|  | UBR1 | NM_174916 | Ubiquitin protein ligase E3 component n-recognin 1 | 0.930 |
|  | VHL | NM_000551 | Von Hippel-Lindau tumor suppressor | 1.351 |
|  | WWP1 | NM_007013 | WW domain containing E3 ubiquitin protein ligase 1 | 0.977 |

**Table S2. Correlation between CHAC2 expression and clinicopathological factors of gastric cancer patients**

| **Clinicopathological parameters** | **N** | **CHAC2 expression** | | **X^2^** | **p-value** |
| --- | --- | --- | --- | --- | --- |
|  |  | **Low (%)** | **High(%)** |  |  |
|  | 99 | 56(56.6) | 43(43.4) |  |  |
| **Gender** | |  |  | 0. 207 | 0.649 |
| Male | 69 | 38(55.1) | 31(44.9) |  |  |
| Female | 30 | 18(60.0) | 12(40.0) |  |  |
| **Age** | |  |  | 0.218 | 0.640 |
| ≥average | 51 | 30(58.8) | 21(41.2) |  |  |
| ﹤average | 48 | 26(54.2) | 22(45.8) |  |  |
| **Histopathological grading** | | |  | 9.550 | **0.002** |
| Well/moderately | 26 | 8(30.8) | 18(69.2) |  |  |
| Poorly | 73 | 48(65.8) | 25(34.2) |  |  |
| **Depth of invasion** | |  |  | 8.386 | **0.039** |
| T1 | 16 | 5(31.3) | 11(68.7) |  |  |
| T2 | 13 | 5(38.5) | 8(61.5) |  |  |
| T3 | 39 | 25(64.1) | 14(35.9) |  |  |
| T4 | 31 | 21(67.7) | 10(32.3) |  |  |
| **Lymph node metastasis** | | |  | 10.187 | **0.017** |
| N0 | 29 | 13(44.8) | 16(55.2) |  |  |
| N1 | 16 | 5(31.3) | 11(68.7) |  |  |
| N2 | 16 | 12(75.0) | 4(25.0) |  |  |
| N3 | 38 | 26(68.4) | 12(31.6) |  |  |
| **Distant metastasis** | |  |  | 2.640 | 0.104 |
| M0 | 83 | 44(53.0) | 39(47.0) |  |  |
| M1 | 16 | 12(75.0) | 4(25.0) |  |  |
| **TNM stages** | |  |  | 11.194 | **0.011** |
| I | 19 | 6(31.6) | 13(68.4) |  |  |
| II | 25 | 11(44.0) | 14(56.0) |  |  |
| III | 39 | 27(69.2) | 12(30.8) |  |  |
| IV | 16 | 12(75.0) | 4(25.0) |  |  |

**Table S3. Correlation between CHAC2 expression and clinicopathological factors of colorectal cancer patients**

| **Clinicopathological parameters** | **N** | **CHAC2 expression** | | **X^2^** | **p-value** |
| --- | --- | --- | --- | --- | --- |
|  |  | **Low (%)** | **High(%)** |  |  |
|  | 131 | 76(58.0) | 55(42.0) |  |  |
| **Gender** | |  |  | 0.439 | 0.507 |
| Male | 79 | 44(55.7) | 35(44.3) |  |  |
| Female | 52 | 32(61.5) | 20(38.5) |  |  |
| **Age** | |  |  | 0.754 | 0.385 |
| ≥average | 63 | 39(61.9) | 24(38.1) |  |  |
| ﹤average | 68 | 37(54.4) | 31(45.6) |  |  |
| **Tumor location** |  |  | | 0.331 | 0.565 |
| Colon | 49 | 30(61.2) | 19(38.8) |  |  |
| Rectum | 82 | 46(56.1) | 36(43.9) |  |  |
| **Histopathological grading** | | |  | 2.106 | 0.147 |
| Well | 86 | 46(53.5) | 40(46.5) |  |  |
| Moderately/poorly | 45 | 30(66.7) | 15(33.3) |  |  |
| **Depth of invasion** | |  |  | 2.857 | 0.091 |
| T1/T2 | 33 | 15(45.5) | 18(54.5) |  |  |
| T3/T4 | 98 | 61(62.2) | 37(37.8) |  |  |
| **Lymph node metastasis** | | |  | 15.296 | **p<0.001** |
| N0 | 69 | 30(43.5) | 39(56.5) |  |  |
| N1/N2 | 62 | 47(75.8) | 15(24.2) |  |  |
| **Distant metastasis** | |  |  | 5.709 | **0.017** |
| M0 | 116 | 63(54.3) | 53(45.7) |  |  |
| M1 | 15 | 13(86.7) | 2(13.3) |  |  |
| **TNM stages** | |  |  | 19.838 | **p<0.001** |
| I | 17 | 4(23.5) | 13(76.5) |  |  |
| II | 49 | 23(46.9) | 26(53.1) |  |  |
| III | 50 | 36(72.0) | 14(28.0) |  |  |
| IV | 15 | 13(86.7) | 2(13.3) |  |  |

**Table S4.** Univariate associations between various factors in patients with gastric cancer and risk of death

| **Characteristics** | **Categories** | **HR** | **95% CI** | **p-value** |
| --- | --- | --- | --- | --- |
| Gender | Male/female | 1.096 | 0.659-1.822 | 0.725 |
| Age | ＜61/≥61 | 1.552 | 0.961-2.507 | 0.073 |
| Histopathological grading | Poorly/moderately/well | 1.388 | 0.956-2.014 | 0.085 |
| Depth of invasion | T1/T2/T3/T4 | 1.651 | 1.280-2.128 | **p<0.001** |
| Lymph node metastasis | N0/N1/N2/N3 | 1.363 | 1.109-1.675 | **0.003** |
| Distant metastasis | M0/M1 | 4.433 | 2.478-7.930 | **p<0.001** |
| TNM stages | I/II/III/IV | 1.765 | 1.347-2.313 | **p<0.001** |
| CHAC2 expression | Low/high | 0.383 | 0.227-0.645 | **p<0.001** |

**Table S5.** Univariate associations between various factors in patients with colorectal cancer and risk of death

| **Characteristics** | **Categories** | **HR** | **95% CI** | **p-value** |
| --- | --- | --- | --- | --- |
| Gender | Male/female | 0.791 | 0.451-1.388 | 0.414 |
| Age | ＜63/≥63 | 0.987 | 0.576-1.692 | 0.962 |
| Tumor location | Rectum/colon | 1.392 | 0.808-2.396 | 0.233 |
| Histopathological grading | Poorly/moderately/well | 1.490 | 1.089-2.040 | **0.013** |
| Depth of invasion | T1/T2/T3/T4 | 0.939 | 0.571-3.287 | 0.788 |
| Lymph node metastasis | N0/N1+N2 | 1.169 | 0.682-2.004 | 0.570 |
| Distant metastasis | M0/M1 | 2.574 | 1.323-5.009 | **0.05** |
| TNM stages | I/II/III/IV | 1.367 | 0.988-1.891 | 0.059 |
| CHAC2 expression | Low/high | 0.363 | 0.194-0.678 | **0.002** |

**Table S6.** Multivariate associations between various factors in patients with gastric cancer and risk of death

| **Characteristics** | **Categories** | **HR** | **95% CI** | **p-value** |
| --- | --- | --- | --- | --- |
| CHAC2 expression | Low/high | 0.548 | 0.312-0.960 | **0.036** |
| TNM stages | I/II/III/IV | 1.555 | 1.165-2.076 | **0.003** |

**Table S7.** Multivariate associations between various factors in patients with colorectal cancer and risk of death

| **Characteristics** | **Categories** | **HR** | **95% CI** | **p-value** |
| --- | --- | --- | --- | --- |
| CHAC2 expression | Low/high | 0.380 | 0.202-0.711 | **0.003** |
| Histopathological grading | Poorly/moderately/well | 1.428 | 1.042-1.957 | **0.027** |

**Table S8.** The correlation between CHAC2 protein expression and XBP-1s, active caspase-3 or Beclin 1 expression in cancer tissues

GC gastric cancer; CRC colorectal cancer.

| **CHAC2 expression in GC** |  | **XBP-1s expression** | | |
| --- | --- | --- | --- | --- |
|  | **N** | **Low (%)** | **High (%)** | **p- value** |
| Low | 56 | 38(67.9) | 18(32.1) | **0.002** |
| High | 43 | 16(37.2) | 27(62.8) |  |
| **CHAC2 expression in GC** |  | **Active caspase-3 expression** | | |
|  | **N** | **Low (%)** | **High (%)** | **p- value** |
| Low | 56 | 49(87.5) | 7(12.5) | **p<0.001** |
| High | 43 | 12(27.9) | 31(72.1) |  |
| **CHAC2 expression in GC** |  | **Beclin 1 expression** | | |
|  | **N** | **Low (%)** | **High (%)** | **p- value** |
| Low | 56 | 51(91.1) | 5(8.9) | **p<0.001** |
| High | 43 | 15(34.9) | 28(65.1) |  |
| **CHAC2 expression in CRC** |  | **XBP-1s expression** | | |
|  | **N** | **Low (%)** | **High (%)** | **p- value** |
| Low | 76 | 63(82.9) | 13(17.1) | **p<0.001** |
| High | 55 | 17(30.9) | 38(69.1) |  |
| **CHAC2 expression in CRC** |  | **Active caspase-3 expression** | | |
|  | **N** | **Low (%)** | **High (%)** | **p- value** |
| Low | 76 | 57(75.0) | 19(25.0) | **p<0.001** |
| High | 55 | 24(43.6) | 31(56.4) |  |
| **CHAC2 expression in CRC** |  | **Beclin 1 expression** | | |
|  | **N** | **Low (%)** | **High (%)** | **p- value** |
| Low | 76 | 59(77.6) | 17(22.4) | **p<0.001** |
| High | 55 | 19(34.5) | 36(65.5) |  |
