# Supplementary figures and images for: CHAC2, downregulated in gastric and colorectal cancers, acted as a tumor suppressor inducing apoptosis and autophagy through unfolded protein response
Source: Cell Death Dis. 2017 Aug 24;8(8):e3009–. doi: 10.1038/cddis.2017.405 (PMC5596586; doi:10.1038/cddis.2017.405)

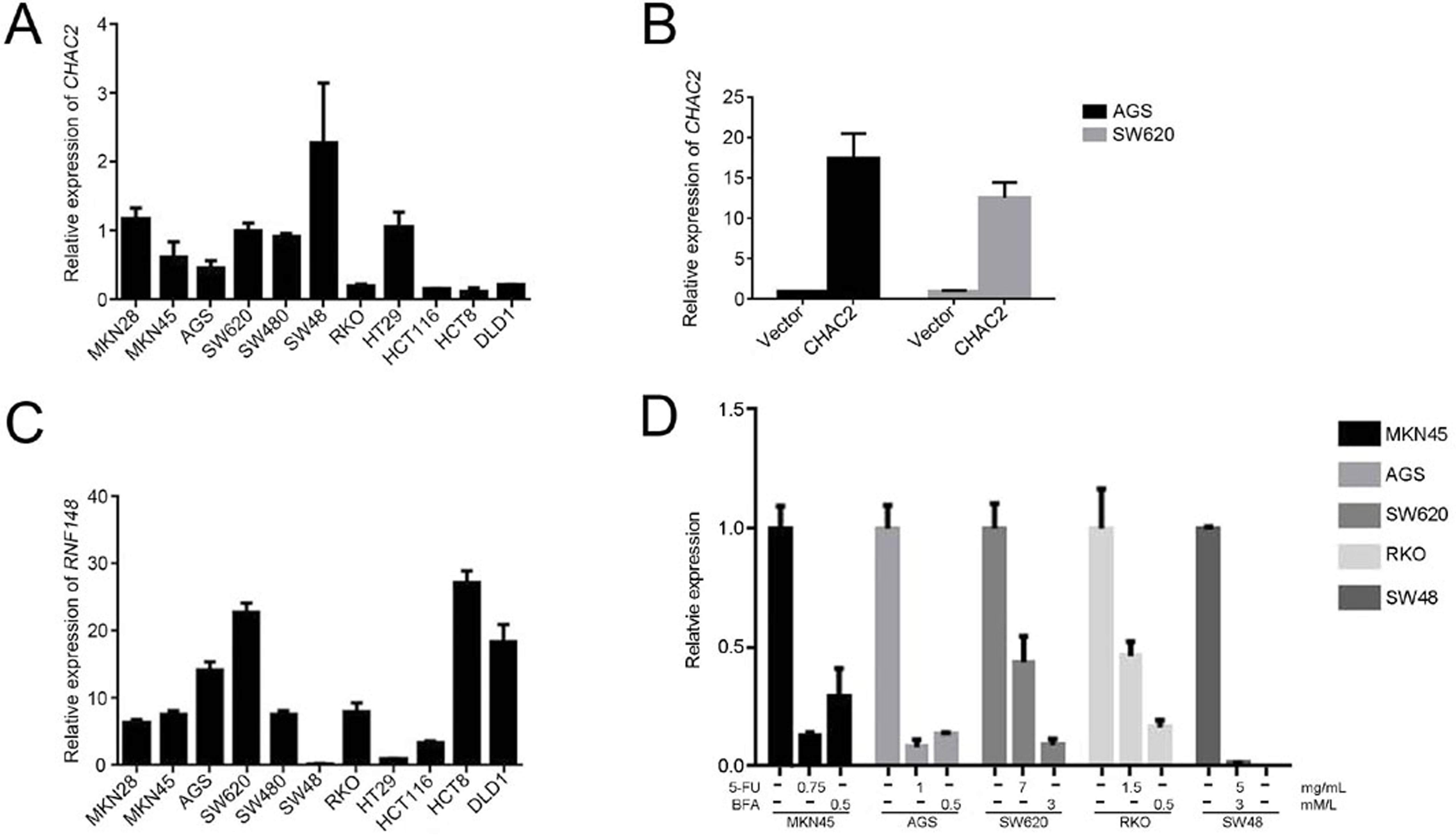

Supplement: Supplementary Figure S1 [file cddis2017405x2.tif]

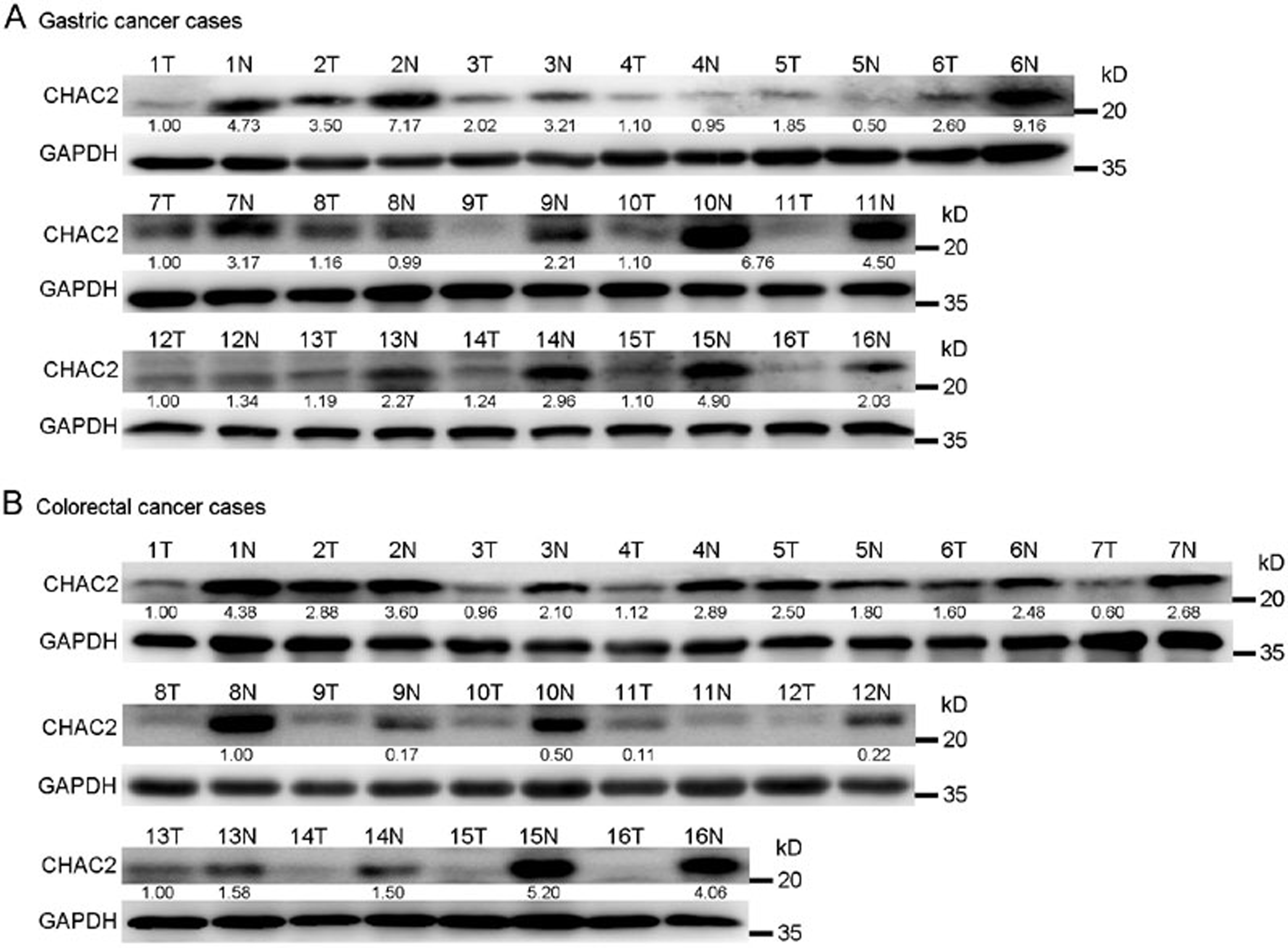

Supplement: Supplementary Figure S2 [file cddis2017405x3.tif]

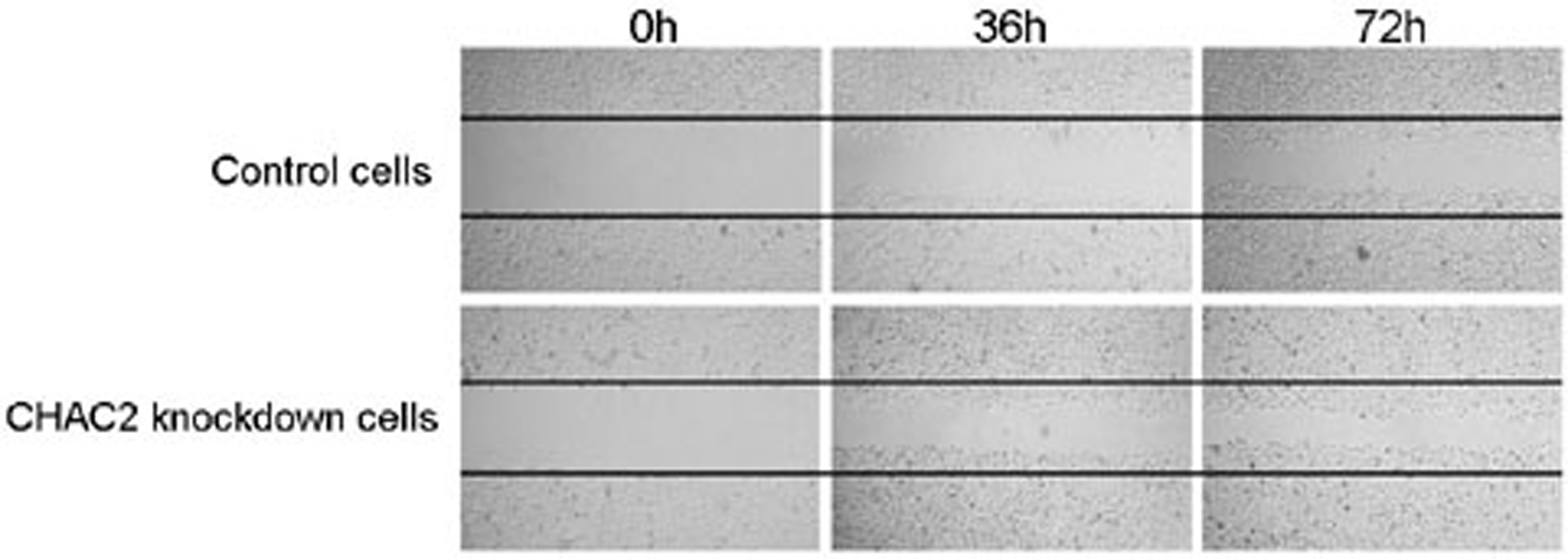

Supplement: Supplementary Figure S3 [file cddis2017405x4.tif]

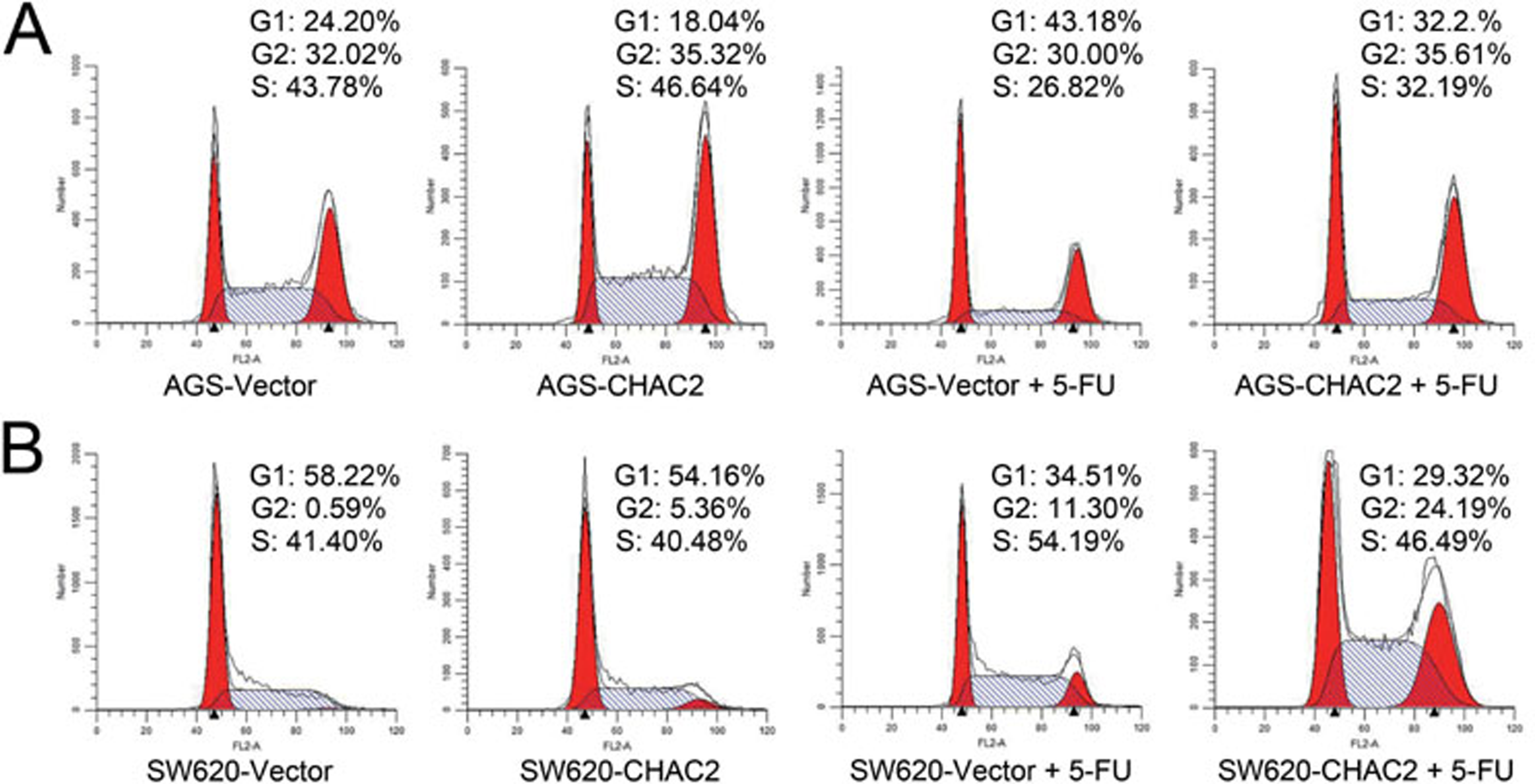

Supplement: Supplementary Figure S4 [file cddis2017405x5.tif]

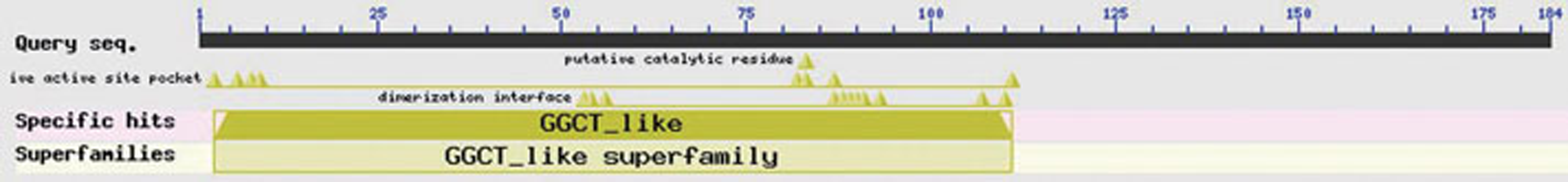

Supplement: Supplementary Figure S5 [file cddis2017405x6.tif]
