# Supplementary material for: CHAC2, downregulated in gastric and colorectal cancers, acted as a tumor suppressor inducing apoptosis and autophagy through unfolded protein response
Source: Cell Death Dis. 2017 Aug 24;8(8):e3009–. doi: 10.1038/cddis.2017.405 (PMC5596586; doi:10.1038/cddis.2017.405)
Supplement: Supplementary Information [file cddis2017405x7.doc]

**Figure S1** Further analysis of CHAC2 or RNF148 expression in gastric and colorectal cancer cell lines by qRT-PCR. **(A)** The expression of CHAC2 mRNA in gastric and colorectal cancer cell lines was determined. **(B)** CHAC2 mRNA level was obviously increased in the CHAC2 stably transfected cells. **(C)** Evaluation of RNF148 mRNA expression in gastric and colorectal cancer cell lines. **(D)** Analysis of RNF148 mRNA expression after treatment of 5-FU or BFA. GAPDH was used as a control in all these experiments.

**Figure S2** CHAC2 protein expression was determined by western blot in tumor tissues and paired non-tumor tissues in 16 primary gastric cancer and 16 colorectal cancer cases. T, tumor tissues; N, paired non-tumor tissues.

**Figure S3** Wound-healing assay results in CHAC2 knockdown SW48 cells and controls. Representative photos were taken at indicated time (original magniﬁcation: 100×).

**Figure S4** The representative results of cell cycle phase distribution of CHAC2-transfected cells and controls. **(A)** The representative results of cell cycle phase distribution of AGS-CHAC2 cells and controls. **(B)** The representative results of cell cycle phase distribution of SW620-CHAC2 cells and controls.

**Figure S5** Coding region and conservative domain structure of CHAC2.
